# Cesarean section without medical indication and risks of childhood allergic disorder, attenuated by breastfeeding

**DOI:** 10.1038/s41598-017-10206-3

**Published:** 2017-08-29

**Authors:** Shuyuan Chu, Yunting Zhang, Yanrui Jiang, Wanqi Sun, Qi Zhu, Bin Wang, Fan Jiang, Jun Zhang

**Affiliations:** 1 MOE-Shanghai Key Laboratory of Children’s Environmental Health, Xinhua Hospital, School of Medicine, Shanghai Jiao Tong University, Shanghai, 200092 China; 2 Laboratory of Respiratory Disease, Affiliated Hospital of Guilin Medical University, Guilin, 541001 China; 3 Child Health Advocacy Institute, Shanghai Children’s Medical Center, School of medicine, Shanghai Jiao Tong University, Shanghai, 200127 China; 4 Department of Developmental and Behavioral Pediatrics, Shanghai Children’s Medical Center, School of medicine, Shanghai Jiao Tong University, Shanghai, 200127 China; 5 School of Public Health, Guilin Medical University, Guilin, 541004 China

## Abstract

Caesarean section (CS) may increase the risk of asthma and allergic diseases in children, but previous studies could not preclude the potential confounding effect of underlying medical indications for CS. We aim to assess the association between CS itself (without indications) and risks of asthma and allergic rhinitis in children. The 2014 Shanghai Children’s Health, Education and Lifestyle Evaluation was a large population-based survey with cluster random probability sampling in 26 primary schools in Shanghai, China, in 2014. The mode of delivery and child history of asthma and allergic rhinitis were reported by parents. We included 12639 children in our analysis. CS without medical indication was associated with an increased risk of childhood asthma. CS without medical indication and CS for fetal complications were associated with increased risks of childhood allergic rhinitis, respectively. In children fed by exclusive breastfeeding or mixed feeding in the first four months after birth, these risks were not significant. In contrast, in children fed by exclusive formula milk, CS was highly significantly associated with childhood asthma and allergic rhinitis. In conclusion, CS without medical indication was associated with increased risks of both childhood asthma and allergic rhinitis. Breastfeeding in early infancy may attenuate these risks.

## Introduction

The global prevalence of childhood asthma has increased in the last 2–3 decades. For instance, the International Study of Asthma and Allergies in Childhood phase III showed an annual increase in prevalence of asthma by 0.12% on average in most of Asia-Pacific region from 2001 to 2010^1^. A similar trend was found in childhood allergic rhinitis^1^. Mounting evidence suggests that cesarean section (CS) may be a risk factor for childhood asthma and allergic rhinitis^2–4^. However, most CS are performed for fetal and/or maternal indications in previous studies^2, 3^. And these indications themselves may be risk factors for childhood allergic disorders. For example, fetal growth restriction and pre-term birth are associated with a high likelihood of both CS and childhood allergic disorders^5, 6^, i.e., the observed associations might be in part due to confounding by indication. Even if such fetal and maternal complications are adjusted in multivariable modeling, residual confounding may still exist, making the validity of the conclusion uncertain. On the other hand, a number of studies have shown that close contact with older siblings and pets in early life may reduce the risk of childhood asthma^7–9^. Therefore, postpartum exposure may mask the association.

China has one of the highest CS rate in the world^10^. CS without medical indication, the majority of which are due to maternal request, is very common, accounting for nearly half of all CS^11^. The one-child-family policy has resulted in single children in most families while having pets is still uncommon in urban areas. Therefore, the urban China provides an ideal setting to study if CS by itself is associated with the risks of childhood allergic disorders.

We carried out this population-based study based on the 2014 Shanghai Children’s Health, Education and Lifestyle Evaluation (the SCHEDULE study) to investigate whether CS without medical indication is associated with the risks of asthma and allergic rhinitis in children.

## Methods

The SCHEDULE study, a population-based cross-sectional survey with cluster random sampling, was conducted in primary schools in Shanghai, China, in June 2014. The Shanghai Municipality was divided into 17 districts and counties, from which seven were randomly selected. Based on a list of all primary schools in these 7 districts, 26 schools were randomly chosen. Pupils from Grade one to five were eligible for this study. In schools with less than 1000 pupils, all of them were eligible; whereas in schools with over 1000 pupils, half of the classes were randomly selected. Once a class was included, all pupils within the class were eligible. A weight was assigned to each subject based on the probability of sampling^12, 13^.

The study protocol was first approved by the Institutional Review Board (Shanghai Children’s Medical Center Research Ethics Committee). All methods were performed in accordance with the declaration of Helsinki. We worked with the Shanghai Education Commission and the selected schools to obtain school permissions for this survey.

A parental informed consent was obtained for each pupil. A self-administered questionnaire was completed by the parents, which included information on parental demographic characteristics, mode of delivery of the index child, diet, physical exercise in a week, psychological behaviors, and history of asthma, allergic rhinitis, autism, attention deficit hyperactivity disorder, dysaudia and diabetes. A questionnaire on the academic performance of each pupil was completed by their teachers. Height and weight of the pupils were measured at the schools. For this analysis, our focus is on CS and childhood asthma and allergic rhinitis.

Information on mode of delivery (CS vs. vaginal delivery) and breastfeeding was reported by parents. We further inquired whether the CS was performed due to woman or family request without medical indication, fetal complications, maternal diseases or pregnant complications, or other reasons. The fetal indications included dystocia, fetal distress, suspected macrosomia or fetal growth restriction, fetal malposition, multiple gestation. The maternal indications consisted of severe maternal chronic diseases or pregnancy complications such as congenital heart disease and severe hypertensive disorders in pregnancy. The other reasons for CS included the history of previous CS, placenta praevia, placental abruption, nuchal cord, premature aging of the placenta, and uterine malformation. Parents were asked if the index child was ever diagnosed by a doctor as having asthma or allergic rhinitis.

We first examined the association between maternal and infant demographic characteristics and mode of delivery. We then explored the associations between CS and the risks of childhood allergic diseases. We further examined the modifiable effect of postpartum breastfeeding on the association between CS and childhood allergic disorders in a stratified analysis. We defined a confounder as a covariant that changed the association between exposure and outcome by 10% or more. The potential confounders were included in the multivariate model. We identified the following potential confounders: maternal education levels (≤9, 10–12, 13–16, or ≥17 years), paternal education levels (≤9, 10–12, 13–16, or ≥17 years), family income (<3.0, 3.0–9.9, 10.0–29.9, or ≥30.0 in ten thousand RMB/year), gender (boy/girl), maternal history of gestational diabetes (including impaired glucose tolerance) (no/yes), and newborn resuscitation (no/yes). Missing data of the confounders were included as a separate category in the analysis. Logistic regression models with SURVEYLOGISTIC procedure and strata with district, school, grade and class in SAS 9.2 (SAS Institute Inc., Cary, North Carolina) were used. The results were presented as odds ratios (OR) and 95% confidence intervals (CI).

## Results

**Figure 1:**
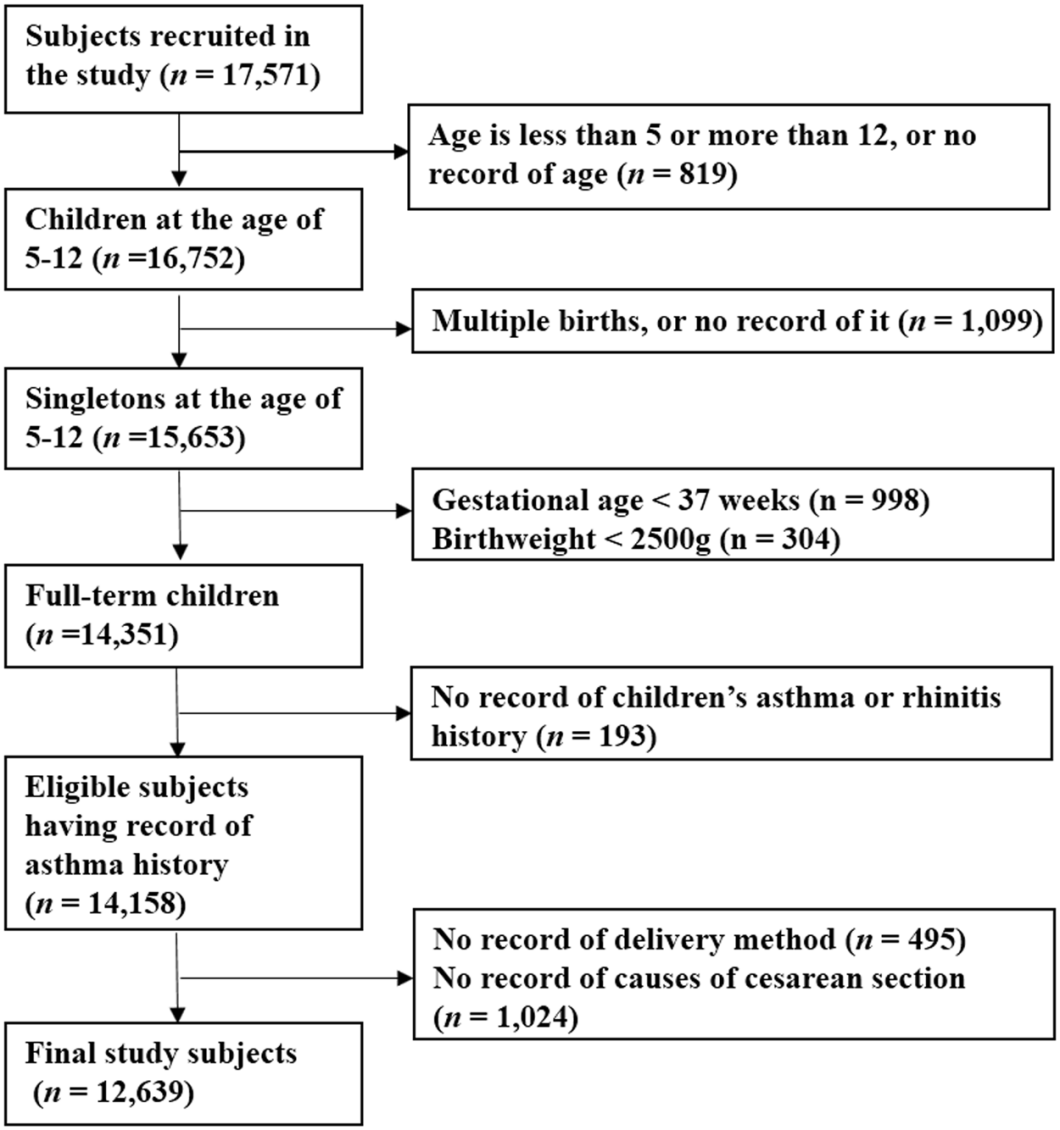
Population flow chart of the population-based study.

A total of 17571 pupils completed this population-based survey (response rate 99%). Figure 1 illustrates the population selection process. The final sample included 12639 pupils. We compared the 1519 excluded subjects with those remained in the analysis with regard to baseline characteristics and the prevalence of asthma and allergic rhinitis (Table S1). They look similar. Among them, 47% were delivered by CS (N = 5962). CS without medical indication, for fetal complications, maternal disorders, and other reasons accounted for 19% (N = 2369), 19% (N = 2424), 2% (N = 267), and 7% (N = 902), respectively (Table 1).

**Table 1:** Demographic, perinatal and child characteristics by mode of delivery in the population-based study.

Characteristics	Vaginal delivery		CS without indications		CS for fetal complications		CS for maternal complications		CS for other reasons	
	No.	%	No.	%	No.	%	No.	%	No.	%
**Gender (boys)**	3541	53	1234	52	1307	54	142	53	490	54
**Age (yrs)**										
5–6	304	5	158	7	130	5	12	4	61	7
7–8	2639	40	1010	43	1051	43	118	44	368	41
9–10	2639	40	896	38	897	37	107	40	342	38
11–12	1095	16	305	13	346	14	30	11	131	15
**Birth weight (g)**										
2500–2999	838	13	257	11	289	12	74	28	103	11
3000–3499	2989	45	1048	44	876	36	103	39	396	44
3500–3999	1848	28	808	34	766	32	78	29	291	32
≥4000	515	8	188	8	452	19	8	3	87	10
**Newborn resuscitation**	25	0	53	2	1	0	23	9	102	11
**Feeding in the first 4 months**										
exclusive breastfeeding	4492	67	1270	54	1373	57	134	50	537	60
mixed feeding	1341	20	684	29	715	29	80	30	224	25
exclusive formula feeding	775	12	399	17	319	13	53	20	134	15
**Passive smoking**										
no	1835	27	595	25	739	30	72	27	237	26
occasionally	3462	52	1218	51	1199	49	129	48	444	49
frequently	1327	20	550	23	472	19	66	25	219	24
**Asthma**	177	3	111	5	92	4	14	5	34	4
**Allergic rhinitis**	427	6	465	20	64	3	64	24	153	17
**Gestational diabetes**	80	1	64	3	86	4	45	17	28	3
**Maternal educational level (yrs)**										
≤9	2650	40	445	19	442	18	53	20	244	27
10–12	1765	26	633	27	631	26	55	21	206	23
13–16	1902	28	1155	49	1223	50	138	52	397	44
≥17	147	2	88	4	98	4	14	5	35	4
**Paternal education level (yrs)**										
≤9	2320	35	393	17	366	15	42	16	219	24
10–12	2021	30	688	29	673	28	70	26	233	26
13–16	1890	28	1112	47	1167	48	131	49	371	41
≥17	292	4	146	6	186	8	18	7	60	7
**Family income (10 thousand RMB/yr)**										
<3.0	687	10	130	5	105	4	20	7	58	6
3.0–9.9	2257	34	655	28	676	28	77	29	268	30
10.0–29.9	1472	22	730	31	745	31	80	30	250	28
≥30.0	2089	31	793	33	842	35	85	32	305	34

CS: caesarean section.

Compared with children delivered by vaginal birth, CS births due to maternal disorders had a higher prevalence of birthweight less than 3000 g, and gestational diabetes. In contrast, children delivered by CS for fetal complications were more likely to be macrosomia. Vaginal births were more likely to receive exclusive breastfeeding in the first 4 month after birth, and their parents’ educational levels and family income levels were lower than those delivered by CS. The prevalence of asthma and allergic rhinitis was 3.4% (428/12 639) and 15.3% (1941/12 639) in our study, respectively, and 1.7% (214/12 639) had both asthma and allergic rhinitis.

**Figure 2:**
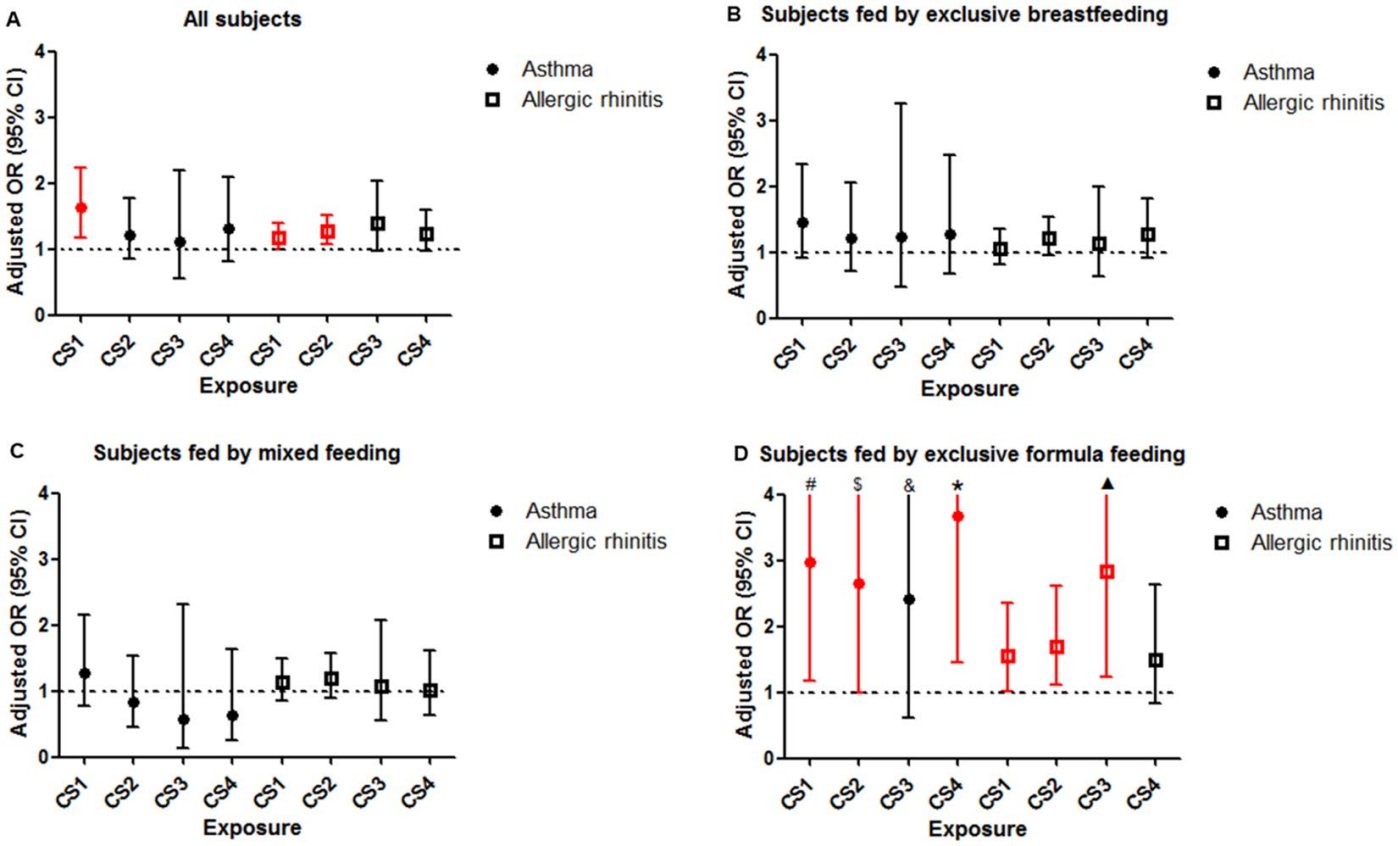
Adjusted and unadjusted relative risk of asthma and allergic rhinitis in children exposed to different modes of delivery. (**A**) All subjects. Asthma: adjusted for maternal education level, paternal education level, maternal history of diabetes in pregnancy; allergic rhinitis: adjusted for maternal education level, paternal education level, maternal history of diabetes in pregnancy. (**B**) Subjects fed by exclusive breastfeeding. Asthma: adjusted for maternal education level, paternal education level, maternal history of diabetes in pregnancy, gender; allergic rhinitis: adjusted for maternal education level, paternal education level, maternal history of diabetes in pregnancy. (**C**) Subjects fed by mixed feeding. Asthma: adjusted for maternal education level, paternal education level, maternal history of diabetes in pregnancy; allergic rhinitis: adjusted for maternal education level, paternal education level, maternal history of diabetes in pregnancy. (**D**) Subjects fed by exclusive formula feeding. Asthma: adjusted for maternal education level, paternal education level, family income, maternal history of diabetes in pregnancy, gender; allergic rhinitis: adjusted for maternal education level, paternal education level, maternal history of diabetes in pregnancy, birth weight, gender. CS1: CS without indications; CS2: CS for fetal complications; CS3: CS for maternal complications; CS4: CS for other reasons. #: upper limit was 7.50; $: upper limit was 6.98; &: upper limit was 9.52; *: upper limit was 9.33; ▲: upper limit was 6.55.

Figure 2 shows that CS without medical indication was associated with an increased risk of childhood asthma after adjusting for potential confounders (adjusted OR = 1.63 [95% CI 1.18–2.24]). CS without medical indication and CS for fetal complications were also associated with increased risks of childhood allergic rhinitis (adjusted OR = 1.18 [95% CI 1.00–1.40] and 1.27 [95% CI 1.08–1.51], respectively) (Fig. 2). These findings were consistent in children with both asthma and allergic rhinitis (Table 2).

**Table 2:** Adjusted and unadjusted relative risks of having history of both asthma and allergic rhinitis in children exposed to different modes of delivery.

Exposure Categories	No.	Unadjusted OR	95% CI	Adjusted OR	95% CI
**All subjects** ^**a**^					
Vaginal delivery	6677	Ref.	\	Ref.	\
CS without indications	2369	2.14	1.35–3.41	1.64	1.01–2.68
CS for fetal complications	2424	1.96	1.23–3.12	1.50	0.93–2.43
CS for maternal disorders	267	3.37	1.53–7.44	2.06	0.90–4.71
CS for other reasons	902	2.01	1.08–3.72	1.71	0.92–3.17
**Subjects fed by exclusive breastfeeding** ^**b**^					
Vaginal delivery	4492	Ref.	\	Ref.	\
CS without indications	1270	1.67	0.80–3.46	1.25	0.58–2.69
CS for fetal complications	1373	1.99	1.06–3.72	1.51	0.80–2.86
CS for maternal disorders	134	2.90	0.88–9.57	2.13	0.60–7.59
CS for other reasons	537	1.63	0.66–4.06	1.36	0.57–3.25
**Subjects fed by mixed feeding** ^**c**^					
Vaginal delivery	1341	Ref.	\	Ref.	\
CS without indications	684	1.49	0.74–3.02	1.26	0.61–2.62
CS for fetal complications	715	1.20	0.56–2.56	0.97	0.44–2.16
CS for maternal disorders	80	1.73	0.46–6.55	1.24	0.27–5.81
CS for other reasons	224	1.15	0.39–3.41	0.98	0.31–3.09
**Subjects fed by exclusive formula feeding** ^**d**^					
Vaginal delivery	775	Ref.	\	Ref.	\
CS without indications	399	11.91	3.96–35.83	10.49	2.63–41.93
CS for fetal complications	319	9.51	2.49–36.39	8.61	2.27–32.64
CS for maternal disorders	53	24.34	4.57–129.56	14.06	2.27–86.95
CS for other reasons	134	17.04	5.04–57.65	15.25	3.94–58.96

^a^Adjusted for maternal education level, paternal education level, family income, maternal history of diabetes in pregnancy.

^b^Adjusted for maternal education level, paternal education level, family income, maternal history of diabetes in pregnancy, birth weight.

^c^Adjusted for maternal education level, paternal education level, maternal history of diabetes in pregnancy.

^d^Adjusted for maternal education level, paternal education level, family income, maternal history of diabetes in pregnancy, birth weight, gender.

CS: caesarean section.

When subjects were stratified by feeding pattern within four months after birth, the risks of having childhood asthma, allergic rhinitis, or both were all substantially higher in children fed by exclusive formula feeding than those fed by exclusive breastfeeding. We examined potential interactions between CS and breastfeeding. No meaningful interactions were found (Tables S2 and S3).

## Discussion

Our study shows that CS without medical indication is associated with increased risks of childhood asthma and allergic rhinitis. CS for fetal complications is also associated with an increased risk of allergic rhinitis in children. This result is consistent with previous studies in general^3, 14, 15^. Breastfeeding may attenuate these risks.

In a recent population-based data-linkage study of 321,287 term singleton first-born offspring in Scotland, United Kingdom, Black *et al*.^14^ found that in comparison with children born vaginally, offspring born by planned CS were at an increased risk of asthma requiring hospital admission (adjusted hazard ratio = 1.22 [95% CI, 1.11–1.34]) and salbutamol inhaler prescription at age 5 years (adjusted hazard ratio = 1.13 [95% CI, 1.01–1.26]). This finding is consistent with that of a meta-analysis, in which CS was associated with a 20% increase in the risk of asthma^3^.

It has been a concern in previous studies that the association between CS and asthma may be due to confounding by indication because the vast majority of CS in those studies were performed for clinical indications. Our studies are the first to specifically examine the association in CS without medical indication. The findings were consistent with previous investigations in general. Although the exact underlying biological mechanism is unclear, it has been hypothesized that fetuses delivered by CS are mainly exposed to microflora that is predominately on maternal skin after birth, but not in maternal vagina^16^. The microbial types and colonization in children delivered by CS may lead to an altered gut microbiota in early life, which may impair natural development of immune system and then promote the development of immune-mediated asthma and allergic disorders^16^. Preliminary evidence suggests that manually exposing newborns delivered by CS to maternal vaginal microbes may partially restore the microbiota of these infants^17^. The protective effect of exclusive breastfeeding on the risks of asthma and allergic rhinitis due to CS in our study, may confirm that microflora-related mechanism, considering that breastfeeding could prevent allergy through regulating infant gut barrier function and microbiota^18^.

Nonetheless, our studies cannot directly prove a causal relationship. Indeed, a sibling analysis using Swedish medical registry data failed to confirm the association between CS and childhood asthma^19^. On the other hand, if gut microflora dysbiosis is causally related to childhood asthma, it is reasonable to question whether the matched sibling design may mask the impact of mode of delivery because the microbial exposure continues after birth. A number of studies have shown that close contact with older siblings and pets may reduce the risk of asthma^7–9^. In Shanghai, China, due to the one-child-family policy, most of our study subjects were single child^20^, and few families have pets at home.

Several limitations of our studies are worth noting. First, information on CS and its indications were self-reported. A previous study demonstrated that the accuracy of maternal recall of CS 3 to 9 years ago was 100%, and maternal recall of severe obstetric complications was also rather reliable^21^. Moreover, the prevalence of CS in our study was 47%, which was consistent with the previous survey in Shanghai (48%)^22^. The rate of CS without medical indications (18%) in our study is also similar to that in a previous report where the rate of CS on maternal request was 20%^11^. Therefore, the self-reported CS and its indications may be reasonably accurate in our study.

Second, asthma and allergic rhinitis in the index children were also reported by the parents. Thus, inaccuracy in the outcome is possible. In our study, the prevalence of asthma was 3.4%, which is lower than 5.81–7.57% reported in previous studies from Shanghai^23, 24^. But the prevalence of allergic rhinitis in our study (15%) was consistent with a previous report (13%)^25^. Discrepancy in asthma prevalence may be partly due to the incompatibility of our study population and the previous ones. In our population-based study, we randomly selected subjects from all 17 districts and counties and included those from suburb and rural areas in Shanghai, whereas the previous studies only selected children from urban areas^23, 24^. Since asthma prevalence in children of rural area in Shanghai is lower than those in urban area (3.7% vs. 6.2%)^26^, the lower prevalence of childhood asthma in our study may be partially expected.

On the other hand, the underestimation of asthma prevalence can’t be completely ruled out, which may be due to underreporting by the parents. It is possible that some parents may not want the school know the child history as this survey was conducted through the school, even though we ensured the parents in the informed consent that the information they provided would be strictly confidential. And we were unable to verify the self-report. On the other hand, we purposefully placed the questions on CS history far apart from those on child disease history in the questionnaire. Thus, we speculate that the misclassification of the outcomes of interest was less likely to be differential. We further controlled for maternal and paternal education and family income in the analysis.

Third, the data on feeding in infancy was recalled by parents. In our study, the prevalence of exclusive breastfeeding was similar to that previously reported in Shanghai, around 50%^27^. Thus, this recall may not be seriously biased.

Finally, our study did not collect data on family history of allergic disorders. Previous study found that children with family history of allergy had a higher risk of asthma than those without family history^28^. Since family history of asthma isn’t related with CS, it may not confound the association between CS and the risks of childhood asthma and allergic rhinitis.

## Conclusions

In conclusion, CS without medical indication is associated with an increased risk of both asthma and allergic rhinitis in children. Our study avoided the challenge of potential residual confounding by CS indications and protective effects by having older siblings and pets^29–31^. In many parts of China, both CS and childhood asthma and allergic disorders are common^22, 32^. CS may have contributed to the increased prevalence of childhood allergic disorders. Fortunately, breastfeeding may attenuate these risks. These findings may have important clinical and public health implications.

## Electronic supplementary material

Table S1-S3
