# Supplementary material for: Cesarean section without medical indication and risks of childhood allergic disorder, attenuated by breastfeeding
Source: Sci Rep. 2017 Aug 29;7:9762. doi: 10.1038/s41598-017-10206-3 (PMC5575109; doi:10.1038/s41598-017-10206-3)
Supplement: Supplementary file 1 — Table S1-S3 [file 41598_2017_10206_MOESM1_ESM.pdf]

Cesarean section without medical indication and risks of childhood allergic disorder, attenuated by breastfeeding

Shuyuan Chu[1,2], Yunting Zhang[3], Yanrui Jiang[4], Wanqi Sun[4], Qi Zhu[4], Bin Wang[1], Fan Jiang[4,*], Jun Zhang [1,5,*]

[1]MOE-Shanghai Key Laboratory of Children's Environmental Health, Xinhua Hospital, School of Medicine, Shanghai Jiao Tong University, Shanghai, 200092,China

[2]Laboratory of Respiratory Disease, Affiliated Hospital of Guilin Medical University, Guilin, 541001, China

[3]Child Health Advocacy Institute, Shanghai Children's Medical Center, School of medicine, Shanghai Jiao Tong University, Shanghai, 200127, China

[4]Department of Developmental and Behavioral Pediatrics, Shanghai Children's Medical Center, School of medicine, Shanghai Jiao Tong University, Shanghai, 200127, China

[5]School of Public Health, Guilin Medical University, Guilin, 541004, China

*Corresponding authors:

Jun Zhang, Xinhua Hospital, School of Medicine, Shanghai Jiao Tong University, Shanghai, 200092, China, Tel: +86 21 2507 8871, Email: zhangjun@xinhuamed.com.cn or

Fan Jiang, Department of Developmental and Behavioral Pediatrics, Shanghai Children's Medical Center, School of medicine, Shanghai Jiao Tong University, Shanghai, 200127, China, Tel: +86 21 3862 6161, Email: fanjiang@shsmu.edu.cn

Table S1. Demographic, perinatal and child characteristics by mode of delivery including subjects excluded for absent information on delivery.

Characteristics	Vaginal delivery		CS including in the study		No record on delivery or without causes of CS	
	No.	%	No.	%	No.	%
**Gender (boys)**	3541	53	3173	53	857	56
**Age (yrs)**						
5-6	304	5	361	6	67	4
7-8	2639	40	2547	43	702	46
9-10	2639	40	2242	38	570	38
11-12	1095	16	812	14	180	12
**Birth weight (g)**						
2500-2999	838	13	723	12	186	12
3000-3499	2989	45	2423	41	629	41
3500-3999	1848	28	1943	33	405	27
≥4000	515	8	735	12	151	10
**Newborn resuscitation**	25	0	179	3	28	2
**Feeding in the first 4 months**						
exclusive breastfeeding	4492	67	3314	56	930	61
mixed feeding	1341	20	1703	29	304	20
exclusive formula feeding	775	12	905	15	230	15
**Passive smoking**						
no	1835	27	1643	28	323	21
occasionally	3462	52	2990	50	837	55
frequently	1327	20	1307	22	354	23
**Asthma**	177	3	251	4	52	3
**Allergic rhinitis**	427	6	746	13	208	14
**Gestational diabetes**	80	1	223	4	32	2

**Maternal educational level (yrs)**						
≤9	2650	40	1184	20	574	38
10-12	1765	26	1525	26	412	27
13-16	1902	28	2913	49	432	28
≥17	147	2	235	4	25	2
**Paternal education level (yrs)**						
≤9	2320	35	1020	17	501	33
10-12	2021	30	1664	28	479	32
13-16	1890	28	2781	47	435	29
≥17	292	4	410	7	50	3
**Family income (10 thousand RMB/yr)**						
＜3.0	687	10	313	5	175	12
3.0-9.9	2257	34	1676	28	466	31
10.0-29.9	1472	22	1805	30	301	20
≥30.0	2089	31	2025	34	59	4

CS: caesarean section

Table S2. Interaction between caesarean section and breastfeeding.

Exposure	Unadjusted estimated beta	P value	Adjusted estimated beta	P value
**Asthma [a]**				
CS	0.29	<0.01	0.21	0.01
Breastfeeding	-0.12	0.70	<0.01	1.00
CS* Breastfeeding	0.41	0.18	0.52	0.09
**Allergic rhinitis [b]**				
CS	0.20	<0.01	0.13	<0.01
Breastfeeding	-0.30	0.05	-0.16	0.30
CS* Breastfeeding	-0.11	0.50	0.02	0.88
**Asthma + Allergic rhinitis [c]**				
CS	0.21	<0.01	0.13	<0.01
Breastfeeding	-0.23	0.10	-0.08	0.56
CS* Breastfeeding	-0.01	0.94	0.13	0.41

a: adjusted for maternal education level, paternal education level, and maternal history of gestational diabetes.

b: adjusted for maternal education level, paternal education level, and maternal history of gestational diabetes.

c: adjusted for maternal education level, paternal education level, family income, and maternal history of gestational diabetes.

CS: caesarean section

Table S3. Interaction between caesarean section and breastfeeding.

Model		Unadjusted estimated OR	Unadjusted estimated 95% CI	Adjusted estimated OR	Adjusted estimated 95% CI
**Asthma [a]**					
OR_{cs}		1.34	1.15-1.57	1.24	1.05-1.46
$OR_{breastfeeding}$		0.88	0.47-1.65	1.00	0.54-1.87
OR_{cs}=1 and $OR_{breastfeeding}$=1	OR_{cs}=1, $OR_{breastfeeding}$=0	2.02	0.96-4.25	2.08	0.98-4.42
	OR_{cs}=0, $OR_{breastfeeding}$=1	1.32	0.39-4.46	1.68	0.50-5.68
**Allergic rhinitis [b]**					
OR_{cs}		1.23	1.14-1.32	1.13	1.05-1.22
$OR_{breastfeeding}$		0.74	0.56-1.00	0.86	0.63-1.15
OR_{cs}=1 and $OR_{breastfeeding}$=1	OR_{cs}=1, $OR_{breastfeeding}$=0	1.10	0.75-1.62	1.16	0.78-1.72
	OR_{cs}=0, $OR_{breastfeeding}$=1	0.67	0.37-1.22	0.88	0.47-1.62
**Asthma + Allergic rhinitis [c]**					
OR_{cs}		1.24	1.15-1.32	1.14	1.06-1.22
$OR_{breastfeeding}$		0.79	0.60-1.05	0.92	0.69-1.22
OR_{cs}=1 and $OR_{breastfeeding}$=1	OR_{cs}=1, $OR_{breastfeeding}$=0	1.22	0.84-1.77	1.30	0.89-1.90
	OR_{cs}=0, $OR_{breastfeeding}$=1	0.78	0.44-1.40	1.05	0.58-1.90

a: adjusted for maternal education level, paternal education level, and maternal history of gestational diabetes.

b: adjusted for maternal education level, paternal education level, and maternal history of gestational diabetes.

c: adjusted for maternal education level, paternal education level, family income, and maternal history of gestational diabetes.

CS: caesarean section
